# Predicting pulmonary function in adolescent idiopathic scoliosis: is a simple 2D radiograph still the winner?

**DOI:** 10.1007/s43390-026-01433-8

**Published:** 2026-05-21

**Authors:** Rafael Garcia de Oliveira, Tarcisio Eloy Pessoa de Barros Filho, Alex de Oliveira Araujo, Raphael Martus Marcon, Arya Varthi, Dominick Tuason, Cicero Ricardo Gomes, Alexandre Fogaça Cristante

**Affiliations:** 1 Faculdade de Medicina, Universidade de São Paulo, São Paulo, Brazil; 2 Yale University, New Haven, USA; 3 Department of Orthopedic Surgery, Pediatrics and Rehabilitation, SARAH Network of Rehabilitation Hospitals, Brasília, Brazil

**Keywords:** Adolescent idiopathic scoliosis, Pulmonary function, 2D-to-3D prediction, Multivariate regression

## Abstract

**Purpose:**

While 3D radiographic parameters are increasingly used to assess adolescent idiopathic scoliosis (AIS), barriers to 3D imaging limit their routine use. 2D-to-3D prediction algorithms have been proposed as a substitute. This study sought to determine if a predicted 3D kyphosis, derived from 2D images, is a more robust predictor of FEV1 in patients with AIS than a traditional 2D analysis.

**Methods:**

A retrospective, cross-sectional review of 259 AIS patients with surgical-range thoracic curves (> 40°) was performed. We built two multivariate linear regression models to predict FEV1. Due to structural multicollinearity, the deformity measures were tested separately. Model A included Main Thoracic Cobb, BMI, and Age. Model B included Predicted 3D T5-T12 Kyphosis, BMI, and Age. The models were compared using the Akaike Information Criterion (AIC) and adjusted.

**Results:**

The cohort (87% female, mean age 15.7 ± 3.3 years) presented with severe deformities (mean Main Thoracic Cobb 68.4° ± 16.5°) and widespread restrictive impairment (71%). Multivariate Analysis revealed that Model A, (2D Main Thoracic Cobb) was statistically superior to Model B (Predicted 3D T5 – T12 Kyphosis) with a lower AIC (−292.5 vs. −265.9) and a higher adjusted *R*^*2*^ (0.241 vs. 0.159). All factors were significant independent predictors.

**Conclusion:**

The predicted 3D-based model was not superior, while a parsimonious 2D-based multivariate model including Main Thoracic Cobb, BMI, and Age explained a significantly larger proportion of the variance in FEV1. This specific 2D-to-3D prediction algorithm is an imperfect proxy and is not a valid substitute for true 3D imaging in predicting pulmonary risk.

## Introduction

Adolescent idiopathic scoliosis (AIS) is a three-dimensional deformity of the spine that presents during the peripubertal years and accounts for the vast majority of idiopathic scoliosis cases [1–3]. While often managed non-operatively, progressive thoracic curves can lead to significant morbidity. There is substantial clinical interest in the relationship between AIS and pulmonary function, as severe, progressive curves can result in thoracic cage distortion and concomitant pulmonary impairment [4–7]. This distortion leads to extrinsic restrictive lung disease, characterized by diminished lung volumes, reduced compliance, and, in rare, severe cases, progression to pulmonary hypertension and *cor pulmonale* [8, 9]. Consequently, preoperative pulmonary function tests (PFTs), particularly FEV1, may be a component of the assessment for AIS patients being considered for surgical correction [10].

Historically, the severity of AIS was quantified on two-dimensional (2D) radiographs, primarily using the coronal Cobb angle. However, the modern understanding of AIS emphasizes its complex 3D nature, involving vertebral rotation in the axial plane and alterations to the sagittal profile [3, 11]. This has led a growing body of literature to investigate 3D radiographic parameters as potentially more accurate predictors of pulmonary dysfunction [12]. Several studies have suggested that sagittal plane parameters, such as thoracic kyphosis, are more consistently correlated with ventilatory decline than coronal measurements alone. For instance, one key study identified thoracic kyphosis as the most consistent predictor of FEV1 (*r*^2^ = 0.087), suggesting its critical role in thoracic cage mechanics [12].

Despite the analytical advantages of 3D imaging, its routine use in the global management of AIS faces significant practical barriers. The requisite low-dose biplanar radiography (EOS) or computed tomography (CT) systems are not universally available, representing a major capital investment [13]. Furthermore, the radiation burden associated with these modalities is a particular concern in an adolescent population that often requires serial imaging over many years [14, 15]. Beyond access, the clinical application of complex 3D parameters requires specialized software and advanced training, creating a "technology gap" between specialized research centers and the typical AIS clinic.

To overcome this limitation, novel statistical and machine-learning models have been developed to generate *predicted* 3D parameters from standard 2D radiographs [16]. These algorithms offer an enticing solution for the AIS population: the analytical power of 3D analysis from a routine, low-dose 2D image. However, a critical question remains. It is unknown if the "noise" and estimation error inherent in these 2D-to-3D *predictions* make them less accurate than the *true* 3D measurements they seek to emulate. More importantly, it is not clear if these predicted 3D values are superior to traditional, reliable 2D measurements in a clinical, multivariate context. This uncertainty leads to our central research question: **in patients with AIS, is a predicted 3D kyphosis, derived from 2D images, a more robust predictor of FEV1 than a traditional 2D analysis?**

## Methods

**Fig. 1:**
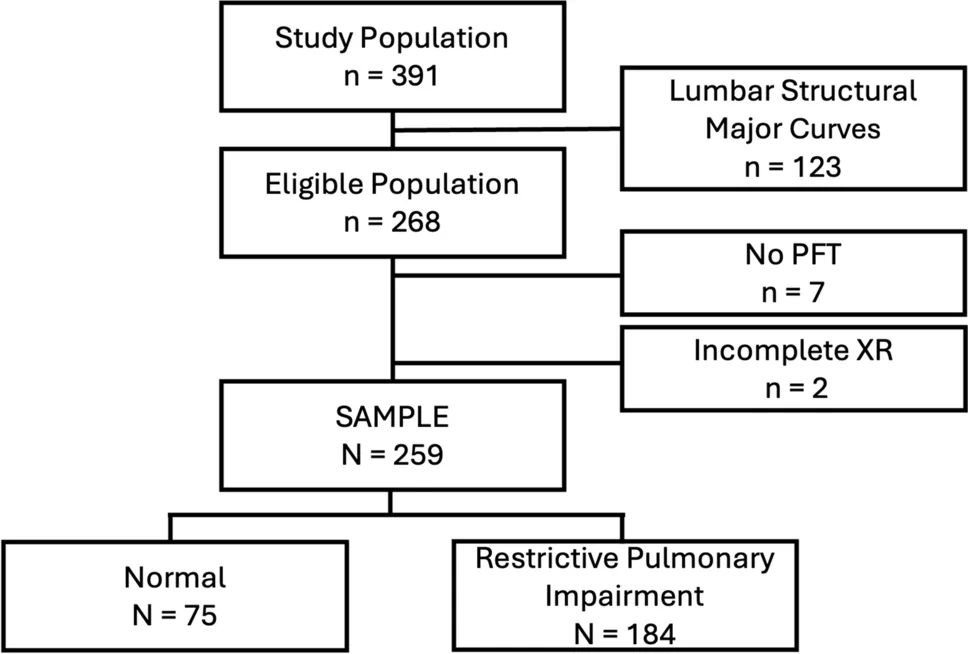
Patient selection flow chart. From an initial study population of 391 patients with adolescent idiopathic scoliosis (AIS) who underwent surgery between 2010 and 2020, 123 were excluded due to the presence of lumbar structural major curves. An additional 7 patients were excluded for missing pulmonary function tests (PFT) and 2 for incomplete radiographs (XR). This resulted in a final sample of 259 patients. Following pulmonary function test, 75 patients (29%) were classified as having normal lung function, while 184 (71%) exhibited restrictive pulmonary impairment

This study was a retrospective, cross-sectional analysis of consecutive patients treated at a single center. Following institutional review board approval, we screened an initial population of 391 patients who underwent surgical correction for adolescent idiopathic scoliosis (AIS) between 2010 and 2020 at Rede Sarah – Brasília, Brazil. As detailed in the patient selection flow chart (Fig. 1), we excluded 123 patients with lumbar structural major curves, 7 without PFTs, and 2 with incomplete radiographs, resulting in a final sample of 259 patients. All data were anonymized and collected from the hospital's electronic record system.

Because scoliosis directly impacts stature and standing height, biological height was estimated using arm span measurements to ensure accurate calculation of BMI and pulmonary predicted values. Demi-span was measured from the tip of the middle finger to the center of the sternal notch. Total arm span was then calculated (demi-span × 2), and biological height was estimated by dividing the span by 1.06 in males and 1.03 in females [17].

Standard two-dimensional (2D) radiographic parameters, including the main thoracic Cobb, proximal thoracic Cobb, and T5-T12 sagittal Cobb were measured by 2 experienced spine surgeons. Three-dimensional (3D) thoracic kyphosis was not measured directly. Instead, a predicted 3D kyphosis was estimated for each patient using the validated regression model published by Parvaresh et al.: Predicted 3D T5 – T12 Kyphosis = 18.1 + (0.81 * 2D T5-T12 Sagittal Cobb)—(0.54 * 2D Main Thoracic Cobb [Coronal]). This model was previously reported to have a strong correlation with true 3D measurements (*R*^*2*^ = 0.84) and a mean absolute error of 5.1° ± 4° in a validation cohort [16].

All PFTs were performed via MasterScope spirometer by a dedicated team, following ATS/ERS guidelines [18]. Tests were repeated three times, using the best valid measurement for FEV1 and FVC[19]. Impairment severity was defined by ATS-ERS criteria [18].

### Statistical analysis

All statistical analyses were performed using R (Version 4.3, R Foundation for Statistical Computing). First, descriptive statistics were calculated; continuous variables were reported as mean and standard deviation (SD), while categorical variables were reported as counts (n) and percentages (%). A univariate analysis was then conducted to identify potential predictors of FEV1. Simple linear regression was used to assess the relationship between FEV1 and continuous variables (e.g., Main Thoracic Cobb, BMI, Age), reporting the Pearson correlation coefficient (*r)*, 95% confidence interval (CI), p-value, and *R*^*2*^. 

To identify independent predictors and control for confounding, multivariate linear regression models were constructed using variables that were significant or clinically relevant in the univariate analysis. We employed a stepwise inclusion methodology to determine the most robust model for FEV1. Candidate variables identified in Table 2, were systematically tested for inclusion; however, their addition did not result in a significant improvement in the model fit, as measured by the Akaike Information Criterion (AIC), nor did they substantially increase the adjusted *R*^*2*^. A single model was not feasible due to structural multicollinearity, as the Predicted 3D Kyphosis formula includes Main Thoracic Cobb. To resolve this dependency, two separate models were built. Model A tested Main Thoracic Cobb (along with BMI and Age), and Model B tested Predicted 3D T5 – T12 Kyphosis (along with BMI and Age). The performance of these final candidate models was formally compared using the Akaike Information Criterion (AIC) and the adjusted R-squared value. A lower AIC and higher adjusted R-squared were considered indicative of a superior model fit. A p-value of 0.05 was considered statistically significant for all tests.

## Results

**Fig. 2:**
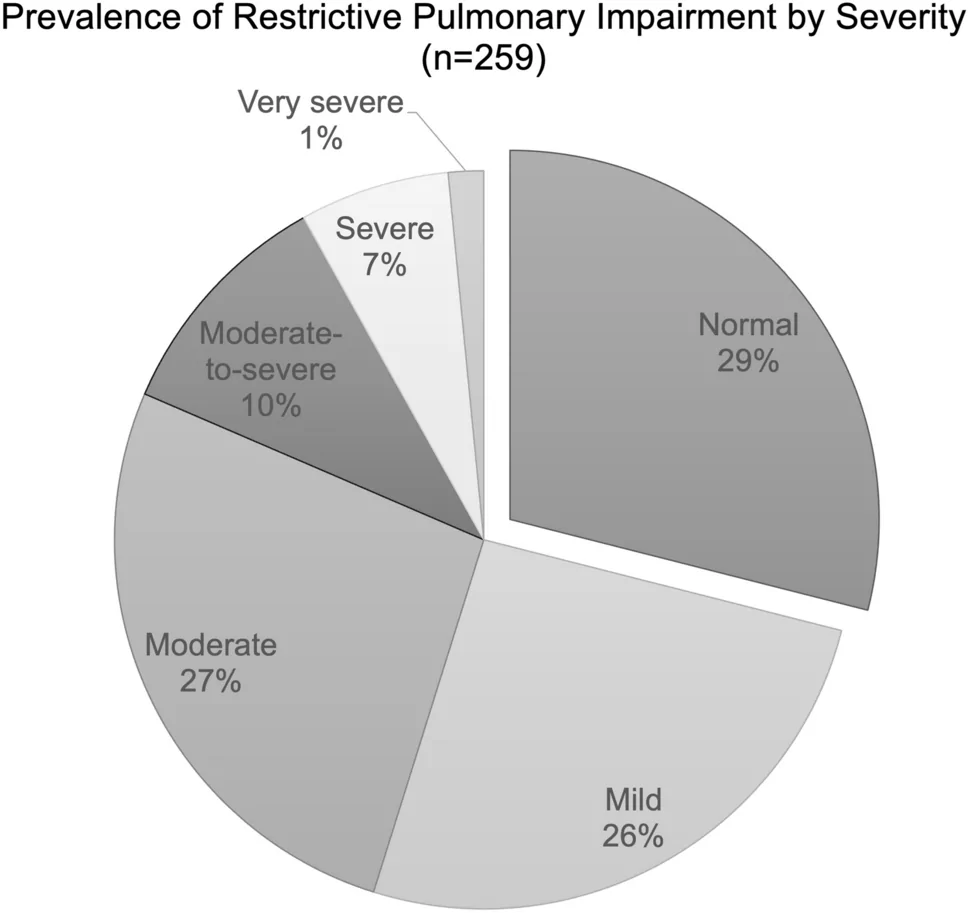
Distribution of respiratory impairment severity in the AIS cohort (n = 259). Patients were classified based on ATS-ERS 2005 criteria [18]. In total, 71% of patients (n = 184) presented with some degree of restrictive impairment. The categories are defined as: Normal (FEV1 > 80% predicted), Mild (FEV1 70–79% predicted), Moderate (FEV1 60–69% predicted), Moderate-to-severe (FEV1 50–59% predicted), Severe (FEV1 35–49% predicted), and Very severe (FEV1 < 35% predicted)

**Fig. 3:**
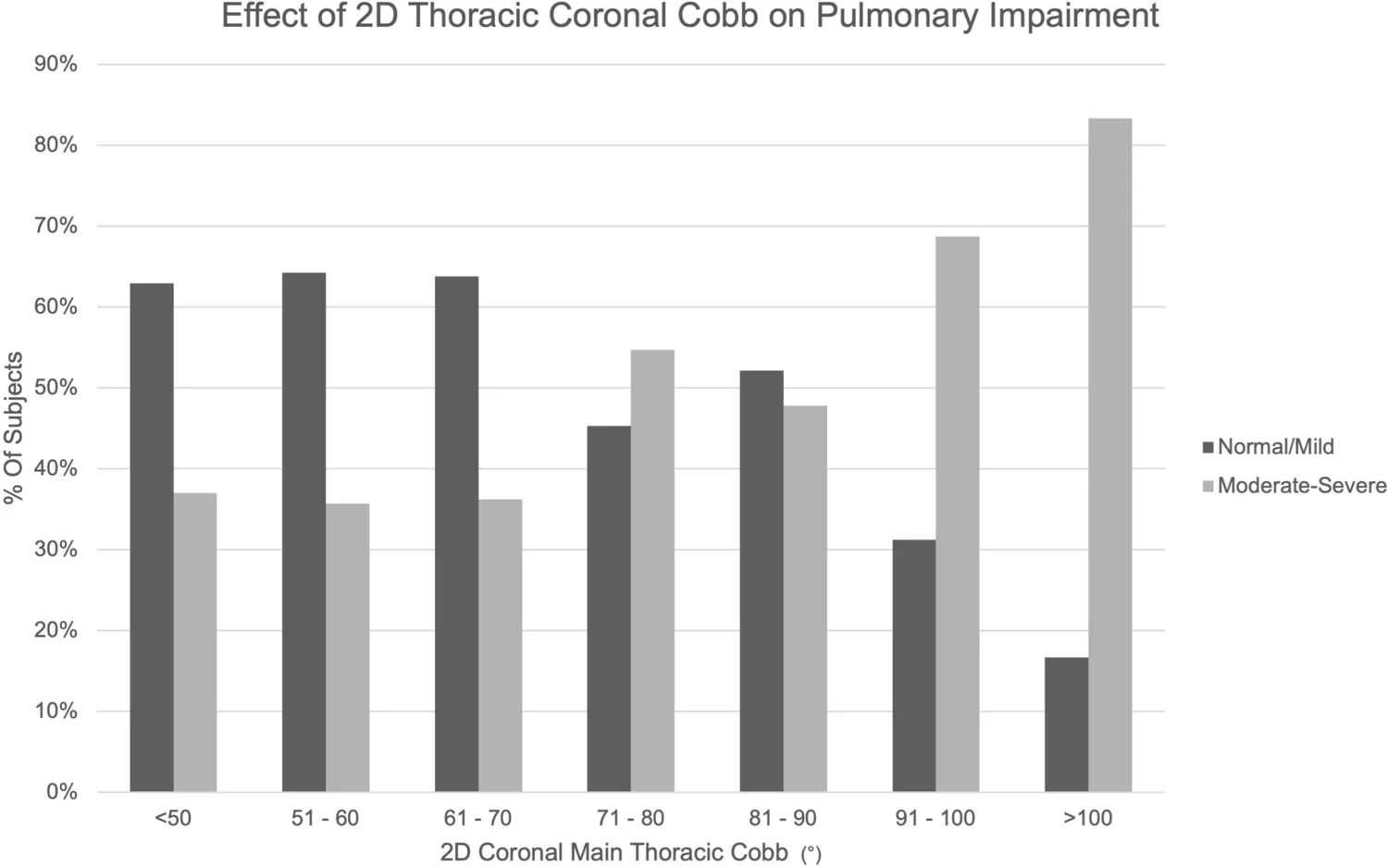
Proportion of patients with Normal/Mild vs. moderate-severe pulmonary impairment, stratified by 2D Coronal thoracic cobb angle. A clear dose–response relationship is visible, with the prevalence of moderate-severe impairment increasing as the coronal curve severity increases

**Fig. 4:**
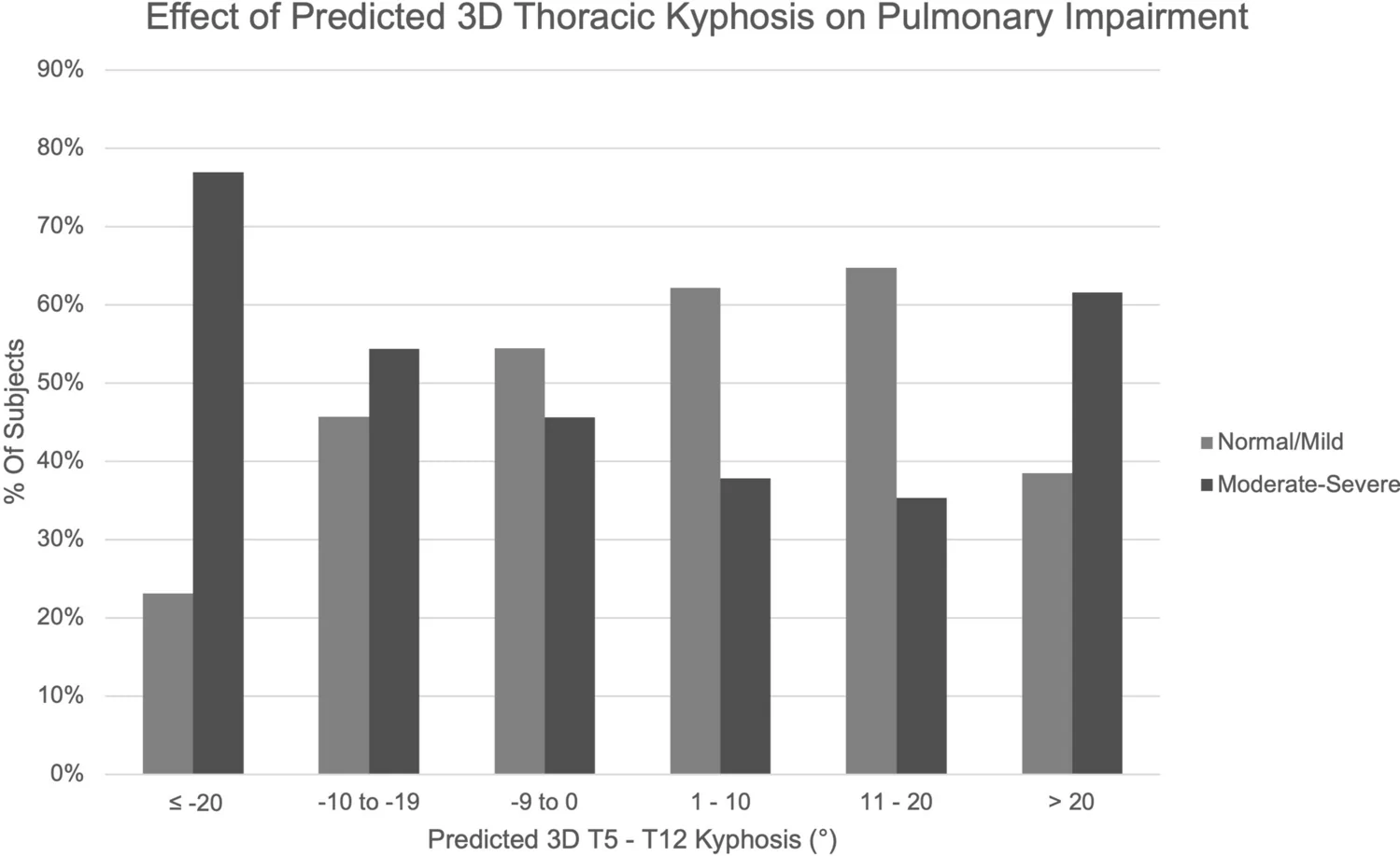
Proportion of patients with Normal/Mild vs. Moderate-Severe pulmonary impairment, stratified by predicted 3D Thoracic Kyphosis. The relationship is non-linear, with the highest prevalence of moderate-severe impairment observed in the most severe hypokyphotic group (− 20°)

A total of 259 patient records were included in the analysis. The cohort's baseline characteristics are presented in Table 1. The pulmonary function data confirmed the expected restrictive pattern for this population. All patients (100%) presented with a normal FEV1/FVC ratio (defined as > 0.7), ruling out obstructive disease. Based on these findings, 184 patients (71%) were classified as having some degree of restrictive respiratory impairment. A detailed breakdown of this impairment severity is visualized in Fig. 2 [18]. A clear dose–response relationship was seen between 2D Coronal Thoracic Cobb and moderate-to-severe pulmonary impairment, which increased with curve magnitude (Fig. 3). When stratified by Predicted 3D T5 – T12 Kyphosis, the highest impairment prevalence (77%) was in the most hypokyphotic group (≤ -−20°), generally decreasing as kyphosis increased (Fig. 4).

**Table 1:** Patient characteristics

Patients’ characteristics (n = 259)	
Age at surgery (yo)	15.7 ± 3.3
Sex (Female)	226 (87%)
BMI	19.8 ± 3.6
Lenke Type 1	73 (28%)
Type 2	60 (23%)
Type 3	67 (26%)
Type 4	59 (23%)
Proximal thoracic cobb (^o^)	30.4 ± 3.6
Main thoracic cobb (^o^)	68.4 ± 16.5
Thoraco-lumbar cobb (^o^)	43.9 ± 15.9
Flexibility of main curve (%)	49.1 ± 16.7
2D T5-T12 kyphosis (^o^)	23.5 ± 15.8
Predicted 3D T5 – T12 kyphosis (^o^)	0.15 ± 12.7
FEV1 (%)^†^	71 ± 0.15
FVC (%)^†^	73 ± 0.16
FEV1/FVC (%)	98 ± 0.1

*BMI* body mass index, *FEV*1 forced expiratory volume in one second, *FVC* forced vital capacity, Predicted 3D Kyphosis = 18.1 + (0.81 * 2D T5-T12 Kyphosis)-(0.54 * 2D Thoracic Cobb). ^†^Data presented as percentage of predicted value. Mean ± Standard Deviation for continuous variables

### Univariate analysis

**Fig. 5:**
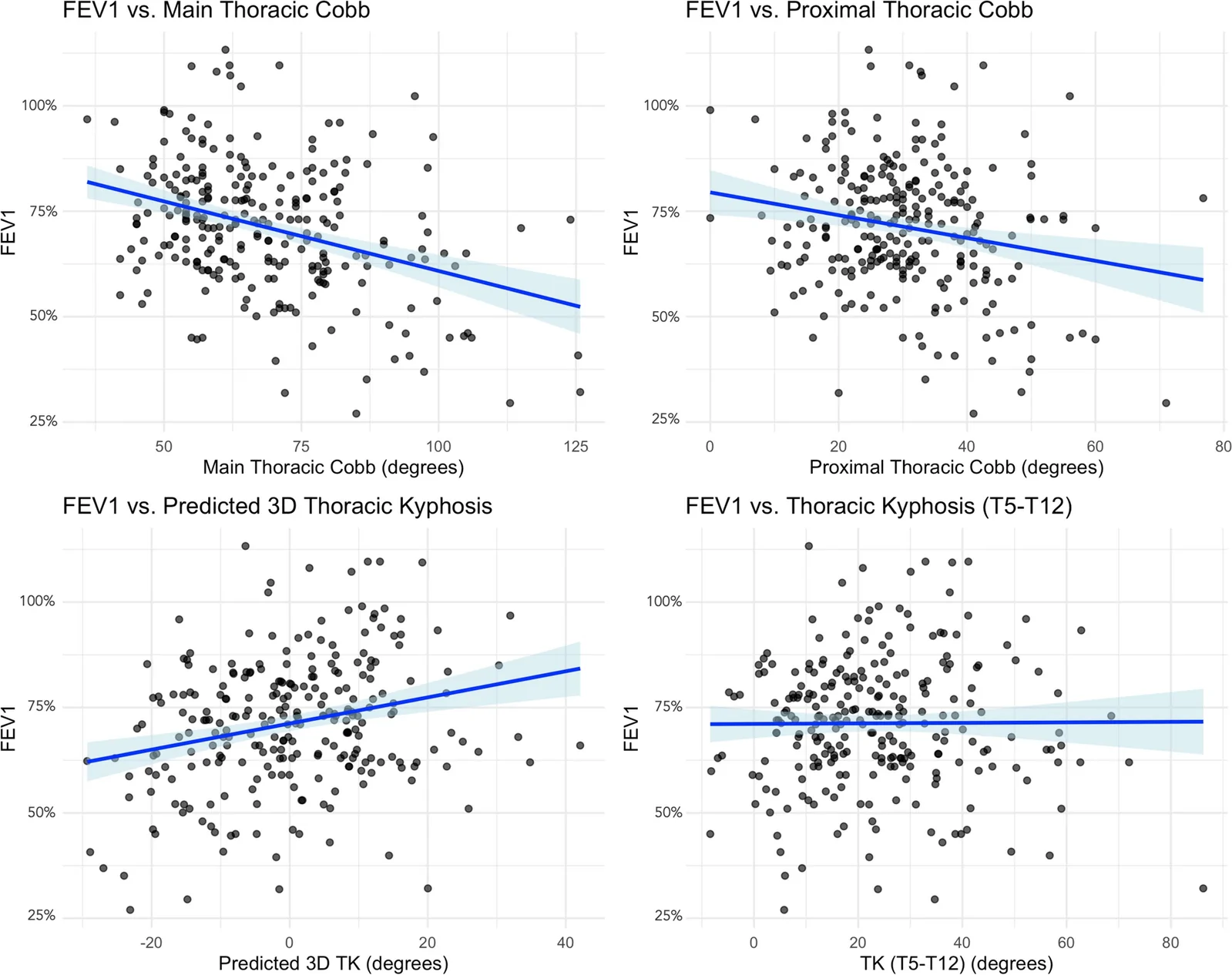
Univariate linear regression analysis of FEV1 against key radiographic deformity parameters. FEV1 is presented as % of the predicted. Analysis shows a significant negative correlation between FEV1 and coronal plane deformities (Main and Proximal Cobb), as well as a significant positive correlation with the Predicted 3D Thoracic Kyphosis. The 2D Thoracic Kyphosis (T5-T12) showed no significant association with FEV1

Univariate analysis showed significant linear relationships between FEV1 and several variables (Fig. 5). Notably, Main Thoracic Cobb demonstrated a significant negative correlation (*r* = − 0.351, p < 0.001), while the Predicted 3D T5 – T12 Kyphosis showed a significant positive correlation (*r* = 0.253, p < 0.001). Other significant predictors included BMI (*r* = 0.275, p < 0.001) and Age (*r* = − 0.156, p = 0.012). Full univariate results are in Table 2.

**Table 2:** Correlation coefficient (r value) of clinical and radiographic variables with pulmonary measurements

Variable	% Predicted FEV1	% Predicted FVC
Age	** − 0.156**	** − 0.166**
BMI	**0.275**	**0.311**
Proximal thoracic cobb (^o^)	** − 0.201**	** − 0.197**
Main thoracic cobb (^o^)	** − 0.351**	** − 0.361**
Thoraco-lumbar cobb (^o^)	0.018	0.014
Flexibility of main curve (%)	** − 0.254**	** − 0.264**
2D T5-T12 kyphosis (^o^)	0.006	** − **0.074
Predicted 3D T5 – T12 kyphosis (^o^)	**0.253**	**0.179**

Bold values represent statistical significance, *p* < 0.05

### Multivariate model analysis

Significant univariate predictors were identified for multivariate analysis. The Main Thoracic Cobb and Predicted 3D T5 – T12 Kyphosis were mathematically dependent. A model including both rendered them non-significant, confirming the statistical necessity of separate models. Therefore, to assess each deformity's independent contribution, two parsimonious models were built, controlling for BMI and Age.

### Model A: 2D-based model

Model A tested the predictive power of the 2D Main Thoracic Cobb. In this model, all three variables were found to be highly significant independent predictors of FEV1. Main Thoracic Cobb maintained a strong negative association (β = −0.0035, *p* < 0.001), as did Age (β = −0.0125, *p* < 0.001), while BMI had a strong positive association (β = 0.0106, *p* < 0.001). The results for Model A are presented in Table 3.

**Table 3:** Results for multivariate: model A (FEV1 ~ main thoracic cobb + BMI + Age) and Model B (FEV1 ~ Predicted 3D T5 – T12 Kyphosis + BMI + Age)

Model A					
	Estimate	SD	*p*	95% CI	
(Intercept)	0.9398	0.0784	p < 0.001	0.7854	1.0942
Main thoracic cobb	− 0.0035	0.0005	p < 0.001	− 0.0046	− 0.0025
BMI	0.0106	0.0024	p < 0.001	0.0059	0.0153
Age	− 0.0125	0.0026	p < 0.001	− 0.0176	− 0.0074
Model B					
(Intercept)	0.6093	0.0619	p < 0.003	0.4875	0.7311
Predicted 3D kyphosis	0.0027	0.0007	0.0002	0.0013	0.0041
BMI	0.0118	0.0025	p < 0.001	0.0069	0.0167
Age	− 0.0083	0.0027	p = 0.0022	− 0.0136	− 0.003

*SD* standard deviation, 95% CI = 95% confidence interval

### Model B: predicted 3D-based model

Model B tested the predictive power of the Predicted 3D T5 – T12 Kyphosis. In this model, all three variables were also significant independent predictors. Predicted 3D T5 – T12 Kyphosis was positively associated with FEV1 (β = 0.0027, *p* = 0.0002), along with BMI (β = 0.0118, *p* < 0.001), while Age was negatively associated (β = −0.0083, *p* = 0.0022). The results for Model B are presented in Table 3.

### Model comparison

The models were formally compared to determine superior fit. Model A was found to be the more robust model, with a lower AIC (− 292.5 vs. −265.9) and a substantially higher adjusted *R*^*2*^ (0.241 vs. 0.159). This indicates the 2D-based model explained a significantly larger proportion of FEV1 variance. The comparison is summarized in Table 4.

**Table 4:** Model fit comparison

Model	AIC	R^2^
Model A (Main Thoracic Cobb)	− 292.5	0.241
Model B (Predicted 3D Kyphosis)	− 265.9	0.159

*AIC* akaike information criterion. A lower AIC and higher adjusted R-squared were considered indicative of a superior model fit

## Discussion

This study supports the established consensus that pulmonary function declines with increasing spinal deformity severity in patients with AIS [4, 5, 7, 7, 12, 20, 21]. We observed a clear, dose–response relationship between the 2D Main Thoracic Cobb and impairment, where the prevalence of moderate-to-severe impairment steadily increased with the coronal curve magnitude (Fig. 3). The sagittal plane's 3D T5 – T12 Kyphosis showed a different pattern, with the highest rate of impairment (77%) seen in the most severe hypokyphotic group (≤ −20°) (Fig. 4). While both deformity measures were significantly correlated with pulmonary function in isolation, the principal and most clinically relevant finding of our study emerged from the multivariate analysis. When comparing predictive models, a parsimonious model based on the traditional 2D Main Thoracic Cobb, BMI, and Age (Model A, *R*^*2*^ = 0.241) was found to be a significantly more robust predictor of FEV1 than the model based on the 3D predicted kyphosis (Model B, *R*^*2*^ = 0.159). This finding suggests that while sagittal profile is clearly important, a foundational 2D coronal measurement, when placed in a proper multivariate context, may retain superior clinical and statistical power over these 2D-to-3D *estimations*.

Our univariate findings are consistent with the work of Newton et al.[6], who identified the 2D thoracic curve magnitude and 2D thoracic hypokyphosis as significant correlates for PFT decline in a large AIS cohort. Our study also strongly supports Yaszay et al.'s key finding regarding the sagittal plane, which was based on *true* 3D reconstructions [12]. Yaszay noted the greatest pulmonary decline was seen in "frankly lordotic" thoracic spines. This is perfectly mirrored in our data, where the most severe hypokyphotic group (≤ −20°) of predicted 3D T5 – T12 Kyphosis demonstrated the highest prevalence (77%) of moderate-to-severe pulmonary impairment. This suggests that even as a 2D-derived prediction, our sagittal parameter successfully captured the same high-risk clinical pattern identified by advanced 3D imaging. The primary divergence, however, lies in the statistical power and clinical utilit*y* of the final predictive models. Yaszay et al. identified true 3D thoracic kyphosis as their most consistent radiographic predictor, yet it yielded a univariate *R*^2^ of only 0.087. In our study, the 2D-to-3D prediction model (Model B) performed similarly, with an *R*^2^ of 0.159. In stark contrast, our Model A, a parsimonious multivariate model based on the traditional 2D main thoracic Cobb combined with Age and BMI, achieved a far more robust *R*^2^ of 0.241. However, despite being statistically superior, the adjusted *R*^2^ indicates that 76% of the variance in FEV1 remains unexplained. This highlights two critical points. First, it supports the conclusion of both Newton and Yaszay that "radiographic measures are not the only factors"; our inclusion of Age and BMI proved essential in building a clinically meaningful model. Second, it suggests that the 2D-to-3D *prediction* algorithm is an imperfect proxy for true 3D, ultimately providing less predictive power than a simpler, 2D-based multivariate model. While this model is a useful supplemental tool, the relationship between spinal deformity and pulmonary function is highly complex and multifactorial, requiring these radiographic metrics to be considered alongside comprehensive clinical assessments.

BMI also emerged as a powerful, independent predictor, with higher BMI associated with better FEV1. This is strongly corroborated by the work of Tung et al. whose analysis demonstrated that a higher BMI was directly correlated with improved pulmonary function, specifically a higher FVC. They showed that BMI acts as an independent contributor, not merely as a secondary effect mediated by the spinal deformity. This consistency suggests that BMI in the AIS population transcends its role as a simple anthropometric measurement, likely serving as a crucial indicator of overall nutritional status and muscle reserve. A higher BMI may reflect a greater capacity for effective respiratory mechanics, conferring an advantage in ventilatory performance. A complementary and non-mutually exclusive hypothesis is that a low BMI may also be a consequence of the pulmonary impairment itself. Patients with significant restrictive disease experience a chronically elevated work of breathing, which increases metabolic demand and can hinder weight gain. In this perspective, a low BMI is not only a predictor but also a result of the pulmonary compromise, creating a vicious cycle: ventilatory restriction leads to low body mass, which in turn compromises respiratory muscle strength and exacerbates the dysfunction.

Age was also identified as a significant, independent negative predictor of FEV1 in our analysis. While the literature traditionally focuses on adolescent cohorts as a single group, our finding that age acts as a continuous risk factor is supported by the work of Johari et al. [4]. They previously noted that pulmonary function damage was more pronounced in older patients and attempted to quantify this, finding that a one-year increase in age correlated with a significant drop in FVC. However, the robustness of their observation was limited by a small sample size (n = 38). The present investigation, with its substantially larger cohort, not only corroborates this association but significantly strengthens it, confirming that Age is a statistically robust, independent predictor of ventilatory decline, even within the adolescent population. This finding has profound clinical and public health implications, particularly in scenarios with restricted access to surgical care. The association between advancing age and restrictive ventilatory disease highlights a "double jeopardy" for patients with neglected treatment: as time passes, not only does the spinal deformity tend to progress, but age *itself* emerges as an independent risk factor for the deterioration of ventilatory function [22, 23].

A key strength and differentiator of the present study is our inclusion cohort. We exclusively analyzed patients with surgical-range deformities (> 40°), whereas much of the established literature includes a broader spectrum of curve severities, often starting at 10° or 20°[6, 12]. It is well-documented that significant pulmonary impairment often only begins to manifest at this 40° threshold [6]. By focusing on this high-risk population, our cohort is not "diluted" by patients with mild curves and normal pulmonary function. This enriched "signal" of impairment within our data likely allowed for a more sensitive and clinically relevant analysis of the relationship between radiographic parameters and ventilatory decline. This may, in part, explain the strong predictive power (*R*^*2*^ = 0.241) of our final model, as we are modeling the relationship precisely in the population for whom it matters most.

### Limitations

This study has important limitations. Most significant is our reliance on a predicted 3D kyphosis, not true 3D reconstructions. Our findings do not refute 3D parameters, but rather show Model A is superior to this specific 2D-to-3D prediction [16]. The error within the algorithm is a confounder. Furthermore, the study's retrospective, cross-sectional design limits our ability to infer causation and track the progression of pulmonary decline over time; we can only report strong statistical associations. Finally, as a single-center study with strict inclusion criteria (right-sided, thoracic AIS > 40°), our results may lack generalizability.

## Conclusion

This study sought to determine if a predicted 3D kyphosis, derived from 2D images, is a more robust predictor of FEV1 in patients with AIS than a traditional 2D analysis. Our findings provide a clear answer: the predicted 3D-based model was not superior. We found that a parsimonious multivariate model based on the 2D Main Thoracic Cobb, BMI, and Age (Model A) was statistically superior, explaining a significantly larger proportion of the variance in FEV1 (*R*^*2*^ = 0.241) than the 3D-prediction-based model (Model B, *R*^*2*^ = 0.159).

While sagittal deformity — particularly hypokyphosis — is clearly associated with pulmonary impairment, the 2D-to-3D prediction algorithm appears to be an imperfect proxy with inferior predictive power. Our findings confirm that deformity alone is an insufficient predictor and that any clinically useful model must incorporate patient-specific factors, such as Age and BMI, which proved to be highly significant, independent predictors. This study suggests that this specific 2D-to-3D prediction model is not a valid substitute for true 3D imaging in predicting FEV. Therefore, this 2D-based approach should be utilized as a supplemental screening tool rather than a definitive diagnostic substitute for true 3D imaging or comprehensive pulmonary function testing.

## Data Availability

The datasets generated and analyzed during the current study are available from the corresponding author on reasonable request.

## References

[1] Cheng JC, Castelein RM, Chu WC, Danielsson AJ, Dobbs MB, Grivas TB et al (2015) Adolescent idiopathic scoliosis. Nat Rev Dis Primers 1(1):15030. 10.1038/nrdp.2015.30 27188385

[2] Wang WJ, Yeung HY, Chu WCW, Tang NLS, Lee KM, Qiu Y et al (2011) Top theories for the etiopathogenesis of adolescent idiopathic scoliosis. J Pediatr Orthop 31(1 Suppl):S14-27. 10.1097/BPO.0b013e3181f73c12 21173615

[3] Newton PO, Fujimori T, Doan J, Reighard FG, Bastrom TP, Misaghi A (2015) Defining the “three-dimensional sagittal plane” in thoracic adolescent idiopathic scoliosis. J Bone Joint Surg Am 97(20):1694–1701. 10.2106/JBJS.O.00148 26491134

[4] Johari J, Sharifudin MA, Ab Rahman A, Omar AS, Abdullah AT, Nor S et al (2016) Relationship between pulmonary function and degree of spinal deformity, location of apical vertebrae and age among adolescent idiopathic scoliosis patients. Singapore Med J 57(1):33–38. 10.11622/smedj.2016009 PMC4728701 26831315

[5] Leong JC, Lu WW, Luk KD, Karlberg EM (1999) Kinematics of the chest cage and spine during breathing in healthy individuals and in patients with adolescent idiopathic scoliosis. Spine 24(13):1310–1315. 10.1097/00007632-199907010-00007 10404572

[6] Newton PO, Faro FD, Gollogly S, Betz RR, Lenke LG, Lowe TG (2005) Results of preoperative pulmonary function testing of adolescents with idiopathic scoliosis. A study of six hundred and thirty-one patients. J Bone Joint Surg Am 87(9):1937–1946. 10.2106/JBJS.D.02209 16140807

[7] Newton PO, Perry A, Bastrom TP, Lenke LG, Betz RR, Clements D et al (2007) Predictors of change in postoperative pulmonary function in adolescent idiopathic scoliosis: a prospective study of 254 patients. Spine 32(17):1875–1882. 10.1097/BRS.0b013e31811eab09 17762296

[8] Payo J, Perez-Grueso FS, Fernandez-Baillo N, Garcia A (2009) Severe restrictive lung disease and vertebral surgery in a pediatric population. Eur Spine J Off Publ Eur Spine Soc Eur Spinal Deform Soc Eur Sect Cerv Spine Res Soc 18(12):1905–1910. 10.1007/s00586-009-1084-8 PMC2899446 19590906

[9] Wen Y, Kai S, Z Y-G, Z G-Q, D T-X (2016) Relationship between lung volume and pulmonary function in patients with adolescent idiopathic scoliosis: computed tomographic-based 3-dimensional volumetric reconstruction of lung parenchyma. Clin Spine Surg 29(8):E396-400. 10.1097/BSD.0000000000000161 27642778

[10] Akazawa T, Kotani T, Sakuma T, Nakayama K, Iijima Y, Torii Y et al (2021) Pulmonary function improves in patients with adolescent idiopathic scoliosis who undergo posterior spinal fusion regardless of thoracoplasty: a mid-term follow-up. Spine Surg Relat Res 5(1):22–27. 10.22603/ssrr.2020-0077 PMC7870321 33575491

[11] Labelle H, Aubin CE, Jackson R, Lenke L, Newton P, Parent S (2011) Seeing the spine in 3D: how will it change what we do? J Pediatr Orthop 31(1 Suppl):S37-45. 10.1097/BPO.0b013e3181fd8801 21173617

[12] Yaszay B, Bastrom TP, Bartley CE, Parent S, Newton PO (2017) The effects of the three-dimensional deformity of adolescent idiopathic scoliosis on pulmonary function. Eur Spine J 26(6):1658–1664. 10.1007/s00586-016-4694-y 27514676

[13] Dubousset J, Charpak G, Dorion I, Skalli W, Lavaste F, Deguise J, Kalifa G, Ferey S (2005) A new 2D and 3D imaging approach to musculoskeletal physiology and pathology with low-dose radiation and the standing position the EOS system. Bull Acad Natl Med 189(2):287–97 16114859

[14] Larson AN, Schueler BA, Dubousset J (2019) Radiation in spine deformity: state-of-the-art reviews. Spine Deform 7(3):386–394. 10.1016/j.jspd.2019.01.003 31053308

[15] Hoffman DA, Lonstein JE, Morin MM, Visscher W, Harris BS, Boice JD (1989) Breast cancer in women with scoliosis exposed to multiple diagnostic x rays. J Natl Cancer Inst 81(17):1307–1312. 10.1093/jnci/81.17.1307 2769783

[16] Parvaresh KC, Osborn EJ, Reighard FG, Doan J, Bastrom TP, Newton PO (2017) Predicting 3D thoracic kyphosis using traditional 2D radiographic measurements in adolescent idiopathic scoliosis. Spine Deform 5(3):159–165. 10.1016/j.jspd.2016.12.002 28449958

[17] Miller MR, Crapo R, Hankinson J, Brusasco V, Burgos F, Casaburi R, Coates A, Enright P, van der Grinten CM, Gustafsson P, Jensen R (2005) General considerations for lung function testing. Eur Respir J 26(1):153–161 10.1183/09031936.05.00034505 15994402

[18] Pellegrino R, Viegi G, Brusasco V, Crapo RO, Burgos F, Casaburi R et al (2005) Interpretative strategies for lung function tests. Eur Respir J 26(5):948–968. 10.1183/09031936.05.00035205 16264058

[19] Pereira CAdeC, Sato T, Rodrigues SC (2007) New reference values for forced spirometry in white adults in Brazil. J Bras Pneumol 33(4):397–406. 10.1590/s1806-37132007000400008 17982531

[20] Lenke LG, Bridwell KH, Blanke K, Baldus C (1995) Analysis of pulmonary function and chest cage dimension changes after thoracoplasty in idiopathic scoliosis. Spine 20(12):1343–1350 7676331

[21] Johnston CE, Richards BS, Sucato DJ, Bridwell KH, Lenke LG, Erickson M (2011) Correlation of preoperative deformity magnitude and pulmonary function tests in adolescent idiopathic scoliosis. Spine 36(14):1096–1102. 10.1097/BRS.0b013e3181f8c931 21270699

[22] Guiroy A, Carazzo C, Camino-Willhuber G, Morales Ciancio A, Remondino R, Nin F et al (2023) Time to surgery for adolescent idiopathic scoliosis: how long does it take? A multicenter study. World Neurosurg X 19:100187. 10.1016/j.wnsx.2023.100187 PMC10070175 37026088

[23] Meves R, Gepedi (Pediatric Scoliosis Deformity Group from Brazilian Spine Society) (2024) Causes of delays in treatment of patients with pediatric scoliosis in the Brazilian Health System. Spine 49(10):741–742. 10.1097/BRS.0000000000004885 38014745

